# Amamentação e comportamentos externalizantes na infância e adolescência em uma coorte de nascimentos

**DOI:** 10.26633/RPSP.2017.142

**Published:** 2017-11-07

**Authors:** Wanêssa Lacerda Poton, Ana Luiza Gonçalves Soares, Ana Maria Baptista Menezes, Fernando César Wehrmeister, Helen Gonçalves

**Affiliations:** 1 Universidade Vila Velha Faculdade de Medicina Vila Velha (ES) Brasil Universidade Vila Velha, Faculdade de Medicina, Vila Velha (ES), Brasil.; 2 University of Bristol, MRC Integrative Epidemiology Unit Population Health Sciences, Bristol Medical School Bristol Reino Unido University of Bristol, MRC Integrative Epidemiology Unit, Population Health Sciences, Bristol Medical School, Bristol, Reino Unido.; 3 Universidade Federal de Pelotas (UFPEL), Faculdade de Medicina Programa de Pós-Graduação em Epidemiologia Pelotas (RS) Brasil Universidade Federal de Pelotas (UFPEL), Faculdade de Medicina, Programa de Pós-Graduação em Epidemiologia, Pelotas (RS), Brasil.

**Keywords:** Aleitamento materno, comportamento, saúde mental, criança, adolescente, Breast feeding, behavior, mental health, child, adolescent, Lactancia materna, conducta, salud mental, niño, adolescente

## Abstract

**Objetivo.:**

*Avaliar a associação entre tempo de amamentação e comportamentos externalizantes na infância e na adolescência*.

**Métodos.:**

*Foram utilizados dados da Coorte de Nascimentos de Pelotas de 1993. As informações sobre amamentação foram coletadas aos 12 meses. O comportamento foi avaliado aos 4 anos pelo instrumento* Child Behavior Checklist *(CBCL) e aos 11 e 15 anos pelo* Strengths and Difficulties Questionnaire *(SDQ), ambos aplicados às mães ou aos responsáveis pela criança. Dos 5 249 participantes da coorte, foram avaliados aqueles com informações completas para amamentação e comportamentos externalizantes: 630 crianças aos 4 anos, 1 227 adolescentes aos 11 anos e 1 199 aos 15 anos. A associação entre duração da amamentação e comportamentos externalizantes foi avaliada por meio de regressão de Poisson com ajuste robusto da variância*.

**Resultados.:**

*Aos 11 anos, após ajuste para fatores de confusão, as crianças que foram amamentadas por pelo menos 6 meses tiveram menor risco de hiperatividade (RR = 0,54; IC95%: 0,32 a 0,91) em comparação às amamentadas por menos de 1 mês. No entanto, aos 4 e 15 anos, a duração da amamentação não esteve associada aos comportamentos externalizantes*.

**Conclusões.:**

*Embora o aleitamento materno por pelo menos 6 meses tenha sido inversamente associado à hiperatividade aos 11 anos, nenhuma associação foi observada aos 4 e aos 15 anos. Novos estudos longitudinais devem considerar outros fatores que influenciam os comportamentos externalizantes, tais como presença do pai no ambiente familiar, violência doméstica e maus-tratos e qualidade da relação mãe-filho*.

## Abstract (en)

**Objective.:**

To assess the association between breastfeeding duration and externalizing behaviors in childhood and adolescence.

**Methods.:**

Data from the 1993 Pelotas Birth Cohort was used. Information on breastfeeding was assessed at 12 months of age. Behavior was assessed at 4 years of age using the Child Behavior Checklist (CBCL), and at ages 11 and 15 years using the Strengths and Difficulties Questionnaire (SDQ), both administered to the mother or caretaker. Of 5 249 cohort participants, those with complete data on breastfeeding and externalizing behaviors were included: 630 children at 4 years of age, 1 277 adolescents at 11 years, and 1 199 at 15 years. Poisson regression with robust variance was used to assess the association between breastfeeding duration and externalizing behaviors.

**Results.:**

After adjustment for confounders, children who were breastfed for least 6 months had lower risk of hyperactivity (RR = 0.54; 95%CI: 0.32-0.91) at age 11 compared to those breastfed for less than 1 month. However, no association was observed between breastfeeding duration and externalizing behaviors at ages 4 and 15.

**Conclusions.:**

Although breastfeeding for at least 6 months was inversely associated with hyperactivity at 11 years of age no association was observed at 4 and 15 years of age. Further longitudinal studies should focus on other aspects influencing externalizing behaviors, such as presence of the father in the family, domestic violence and abuse, and the quality of mother-child relationship.

## RESUMEN

**Objetivo.:**

Evaluar la relación existente entre la duración del amamantamiento y los patrones de comportamiento externalizante en la infancia y la adolescencia.

**Métodos.:**

Se utilizaron datos de la cohorte de nacimientos de Pelotas de 1993. La información sobre el amamantamiento se recolectó a los 12 meses. El comportamiento se evaluó a los 4 años con la lista de comprobación del comportamiento del niño (Child Behavior Checklist, CBCL) y a los 11 y 15 años con el cuestionario de fortalezas y dificultades (Strengths and Difficulties Questionnaire, SDQ), ambos aplicados a las madres o a los responsables del niño. De los 5 249 participantes de la cohorte, se evaluaron los grupos respecto de los cuales había información completa sobre el amamantamiento y los patrones de comportamiento externalizante: 630 niños a los 4 años, 1 227 adolescentes a los 11 años y 1 199 a los 15 años. La relación entre el período de amamantamiento y los patrones de comportamiento externalizante se evaluó por medio de la regresión de Poisson con un ajuste robusto de la varianza.

**Resultados.:**

A los 11 años, tras el ajuste realizado para tener en cuenta los factores de confusión, los niños amamantadas por un mínimo de 6 meses tuvieron un menor riesgo de hiperactividad (RR = 0,54; IC 95%: de 0,32 a 0,91) en comparación con los amamantados por menos de un mes. Sin embargo, a los 4 y 15 años, la duración del amamantamiento no guardó relación alguna con los patrones de comportamiento externalizante.

**Conclusiones.:**

Aunque la lactancia materna por un mínimo de 6 meses haya guardado una relación inversamente proporcional con la hiperactividad a los 11 años, no se observó ninguna relación a los 4 y a los 15 años. En nuevos estudios longitudinales es preciso considerar otros factores que influyen en los patrones de comportamiento externalizante, como la presencia del padre en el ámbito familiar, la violencia doméstica, el maltrato y la calidad de la relación entre la madre y el niño.

Estimativas apontam que uma em cada quatro a cinco crianças e adolescentes em todo o mundo apresenta algum tipo de transtorno mental (1). Também há evidências de que entre 13% e 20% das crianças terão algum transtorno ou problema de saúde mental na vida (2).

Na infância, os comportamentos externalizantes – caracterizados por oposição, hiperatividade, agressão, desafio, impulsividade e manifestações antissociais (3, 4) – são os mais problemáticos. Por sua relação com o desenvolvimento de comportamentos violentos e criminalidade durante a adolescência ou idade adulta, os comportamentos externalizantes são considerados, atualmente, uma questão de saúde pública (5) e têm forte influência no desenvolvimento emocional e social da criança. Consequentemente, podem ter impacto na adolescência e vida adulta, comprometendo o bem-estar, a capacidade intelectual e as escolhas do indivíduo, alterando seu desempenho individual, familiar e social (2, 3, 6).

Estudos mostram que o aleitamento materno reduz a mortalidade infantil e o desenvolvimento de infecções respiratórias e gastrointestinais, além de estar associado a maiores escores de desenvolvimento cognitivo (7–9). Pesquisadores também têm investigado sua associação com diversos problemas de comportamento (10–19). Entretanto, os poucos estudos disponíveis que investigam a relação do aleitamento materno com os comportamentos externalizantes na infância (10–15, 17, 18) e na adolescência (10, 16, 19) produziram resultados inconsistentes: alguns demonstram que crianças amamentadas, em geral por períodos superiores a 4 ou 6 meses, apresentam menos problemas associados a comportamentos externalizantes na infância (10–12, 18) e na adolescência (10, 19); outros estudos, por sua vez, não detectaram essa associação em nenhuma das fases (13–17).

Considerando esse cenário, o objetivo do presente estudo foi avaliar a relação entre o tempo de aleitamento materno e os comportamentos externalizantes em participantes de uma coorte de nascimentos aos 4 anos (infância) e aos 11 e 15 anos (adolescência).

## MATERIAIS E MÉTODOS

O presente estudo utilizou dados da infância (perinatal, 12 meses e 4 anos) e adolescência (11 e 15 anos) de uma coorte estabelecida em 1993 na cidade de Pelotas, estado do Rio Grande do Sul, Brasil. Em 1993, foram identificadas todas as 5 265 crianças de famílias residentes na zona urbana e nascidas em maternidades de Pelotas. Dessas, 5 249 (99,7%) mães aceitaram que seus filhos participassem da coorte. As mães foram entrevistadas após o nascimento, por profissionais treinados, que utilizaram um questionário padronizado para coletar informações socioeconômicas, demográficas e comportamentais e indagar sobre morbidades, história reprodutiva e assistência pré-natal e ao parto (20, 21). As crianças foram medidas, pesadas e examinadas com instrumentos de precisão (20, 21). Desde então, os participantes têm sido acompanhados em diferentes momentos. Informações metodológicas referentes a cada acompanhamento estão disponíveis em outras publicações (20, 21).

Os acompanhamentos realizados aos 12 meses e aos 4 anos incluíram todas as crianças nascidas com peso inferior a 2 500 g, juntamente com uma amostra sistemática de 20% das demais crianças da coorte, compondo um total de 1 460 participantes. Desse grupo, 1 363 (93,4%) participaram do acompanhamento aos 12 meses e 1 273 (87,2%) participaram aos 4 anos. Na adolescência, aos 11 e 15 anos, todos os participantes da coorte foram convidados a participar dos acompanhamentos, com participação de 4 452 adolescentes (87,5%) aos 11 anos e de 4 349 adolescentes (85,7%) aos 15 anos (21).

No presente estudo, apenas indivíduos com informações completas sobre aleitamento materno e comportamentos externalizantes foram analisados em cada idade de interesse: 630 crianças aos 4 anos, 1 227 adolescentes aos 11 anos e 1 199 aos 15 anos.

### Aleitamento materno

As informações sobre amamentação foram obtidas no acompanhamento dos 12 meses. A idade do desmame foi definida como a interrupção total da amamentação. A duração da amamentação foi coletada de forma contínua e categorizada em < 1 mês; 1 a 2,9 meses; 3 a 5 meses; 9 meses; e ≥ 6 meses. Tendo em vista que somente um pequeno percentual de crianças nunca foi amamentado (3,8%), essas foram agrupadas àquelas amamentadas por < 1 mês. Devido à baixa proporção de crianças amamentadas exclusivamente (7%), o aleitamento materno exclusivo não foi avaliado neste estudo.

### Comportamentos externalizantes

No estudo de coorte, os instrumentos de avaliação do comportamento foram aplicados aos 4, 11 e 15 anos aos responsáveis pelos participantes (mais de 90% eram mães em todos os acompanhamentos). Aos 4 anos, foi aplicado o Inventário de Comportamentos da Infância e da Adolescência (versão brasileira da *Child Behavior Checklist*, CBCL) (4, 22). Aos 11 e 15 anos, foi aplicado o Questionário de Capacidades e Dificuldades (versão brasileira do *Strengths and Difficulties Questionnaire*, SDQ) (23, 24).

O CBCL avalia o grau de competência social e a presença e intensidade dos problemas de comportamento em crianças de 4 a 18 anos, contendo 138 itens agrupados em 12 escalas para avaliar nove tipos de comportamento: retraimento; queixas somáticas; ansiedade/depressão; problemas com o contato social; problemas com o pensamento; problemas com a atenção; problemas sexuais; comportamento delinquente; e comportamento agressivo (22). Para este estudo, os comportamentos delinquente e agressivo foram utilizados. O somatório de ambas as escalas foi considerado para determinar a presença de comportamentos externalizantes.

O SDQ é adequado para utilização em crianças e adolescentes de 4 a 16 anos de idade (24). O instrumento possui 25 itens agrupados em cinco escalas: hiperatividade, problemas emocionais, problemas de conduta, problemas com os colegas e comportamento pró-social. Cada escala possui um escore de classificação singular, que é obtido somando-se as pontuações dos itens que a compõem, podendo variar de 0 a 10. As escalas do SDQ investigadas neste estudo foram hiperatividade e problemas de conduta, que, quando agrupadas, compreendem os comportamento externalizantes.

Os comportamentos foram agrupados em duas categorias: normal (percentil < 90) e anormal (percentil ≥ 90) da distribuição dos escores do CBCL e do SDQ. Este ponto de corte foi anteriormente identificado como diferenciador dos escores normal e anormal em população de baixo risco (25).

### Variáveis de confusão

As variáveis de confusão, medidas no período perinatal, foram: renda familiar mensal (quintis), escolaridade materna em anos de estudo (0 a 4; 5 a 8; 9 a 11; ≥ 12 anos), idade da mãe em anos (< 20; 20 a 24; 25 a 29; 30 a 34; ≥ 35 anos), presença de companheiro ou marido vivendo com a família (sim; não), tabagismo durante a gestação (sim; não), consumo de álcool durante a gestação (sim; não), idade gestacional em semanas (< 37; ≥ 37 semanas), sexo (masculino; feminino) e peso de nascimento em gramas (< 2 500 g; ≥ 2 500 g).

Embora a saúde mental materna seja um importante fator de confusão na relação entre aleitamento materno e problemas de comportamento (26, 27), os dados disponíveis sobre saúde mental materna foram mensurados apenas aos 11 anos; nesse momento, a variável saúde mental materna passa a ser mediadora ou desfecho da associação de interesse e não mais um possível fator de confusão.

### Análise estatística

Inicialmente, as características socioeconômicas, demográficas e de saúde dos participantes nos acompanhamentos dos 4, 11 e 15 anos foram comparadas à coorte original. A relação entre a amamentação e as variáveis de confusão foi avaliada por meio do teste do qui-quadrado (x2) de heterogeneidade ou de tendência linear, conforme o caso. Para a comparação entre as médias das pontuações dos comportamentos externalizantes, conforme as variáveis de confusão, foi empregada a análise de variância (ANOVA).

Para avaliar a associação entre tempo de amamentação e comportamentos externalizantes utilizou-se regressão de Poisson com ajuste robusto da variância nas análises brutas e ajustadas. A multicolinearidade foi testada utilizando o fator de inflação da variância, sendo considerada presente quando maior que 10. Em razão de o estudo utilizar subamostras nas quais houve superestimação dos nascidos com baixo peso (12 meses e 4 anos), todas as análises foram ponderadas. Foi investigada possível interação com o peso de nascimento e a idade gestacional na relação entre amamentação e comportamentos externalizantes, a qual não se mostrou evidente (*P* > 0,05). O efeito dose-resposta do tempo de aleitamento materno sobre os problemas de comportamento foi avaliado utilizando-se o tempo de aleitamento materno como variável categórica e contínua.

As análises foram conduzidas no Stata versão 13.0. O estudo foi aprovado pelo Comitê de Ética em Pesquisa com Seres Humanos da Faculdade de Medicina da Universidade Federal de Pelotas (no 4.06.1.095). O termo de consentimento livre e esclarecido (TCLE) foi fornecido à mãe ou responsável nas visitas de seguimento e aos adolescentes nos acompanhamentos dos 11 e 15 anos.

## RESULTADOS

Os participantes da coorte eram, em sua maioria, do sexo feminino (50,3%) e nascidos com pelo menos 37 semanas de gestação (88,2%) e peso normal (90,3%) (tabela 1). Ao comparar as características dos participantes nos acompanhamentos aos 4, 11 e 15 anos com a coorte original, observa-se que, com exceção da idade materna dos participantes aos 4 anos – cujas mães eram mais velhas (*P* = 0,009) –, não houve diferenças nas amostras em relação às demais variáveis. Cerca de 60% dos participantes foram amamentados por 3 meses ou mais, sendo que 35,2% receberam leite materno por pelo menos 6 meses (tabela 1).

A descrição do tempo de aleitamento materno segundo variáveis socioeconômicas, demográficas e comportamentais é apresentada na tabela 2. O tempo de aleitamento materno foi semelhante conforme fatores socioeconômicos; porém, foi maior nas mulheres com maior idade (*P* < 0,001), que não fumaram durante a gestação (*P* = 0,005) e cujo bebê nasceu com peso normal (*P* < 0,001) e a termo (*P* < 0,001).

A tabela 3 apresenta a média do escore dos comportamentos externalizantes conforme as variáveis de confusão e o tempo de aleitamento materno. Aos 4 anos, a média do escore dos comportamentos externalizantes foi maior entre crianças cujas mães não tinham a presença do marido ou companheiro vivendo com a família (*P* = 0,002) e que fumaram durante a gravidez (*P* < 0,001). Aos 11 e 15 anos, a média do escore para os comportamentos externalizantes foi maior entre adolescentes pertencentes a famílias de menor renda, cujas mães tinham menor escolaridade e menor idade, não possuíam parceiro/marido morando em casa e que haviam fumado (*P* < 0,001) ou consumido bebida alcóolica durante a gravidez (*P* < 0,05). Em ambas idades (11 e 15 anos), os meninos apresentaram maiores médias no escore para os comportamento externalizantes. Relação inversa entre o tempo de aleitamento materno e os comportamentos externalizantes foi observada somente aos 11 anos (*P* = 0,009).

Escore anormal para comportamentos externalizantes foi encontrado em 9,6% das crianças aos 4 anos de idade e em 10,4% dos adolescentes em ambos os acompanhamentos aos 11 e 15 anos (dados não apresentados em tabela). A tabela 4 sintetiza os resultados das análises bruta e ajustada para a relação entre tempo de aleitamento materno e problemas de comportamento externalizantes. Aos 4 anos, tanto na análise bruta quanto na análise ajustada, não foi encontrada associação entre tempo de amamentação e comportamentos externalizantes, comportamento de quebrar regras e comportamento agressivo. Aos 11 anos, adolescentes amamentados por pelo menos 6 meses apresentaram menor risco para hiperatividade, mesmo após ajuste para fatores de confusão (RR = 0,54; IC95%: 0,32 a 0,91), em comparação aos amamentados por menos de 1 mês. No entanto, não foi observado efeito dose-resposta entre o tempo de aleitamento materno e menor risco de hiperatividade (*P* = 0,143). Aos 15 anos, embora os resultados tenham apontado na mesma direção do observado aos 11 anos, o aleitamento materno não se mostrou associado a nenhum dos comportamentos externalizantes avaliados.

## DISCUSSÃO

Este estudo evidenciou menor risco de hiperatividade aos 11 anos em adolescentes amamentados por pelo menos 6 meses quando comparados aos amamentados por menos de 1 mês. Por outro lado, aos 4 e aos 15 anos, apesar de os resultados irem na mesma direção do observado aos 11 anos, nenhuma associação foi observada entre aleitamento materno e comportamentos externalizantes.

É possível que a associação observada entre aleitamento materno e comportamentos externalizantes aos 11 anos neste estudo seja proveniente de confusão residual, uma vez que não foi observado efeito dose-resposta neste e em outros estudos (12, 28), nem a persistência desse efeito protetor em diferentes faixas etárias.

Um estudo longitudinal realizado na Austrália com 1 707 crianças avaliadas usando o CBCL também não detectou diferenças em comportamentos externalizantes aos 5 anos nas crianças amamentadas e não amamentadas, embora tenha observado melhor saúde mental, de forma geral, naquelas amamentadas por mais tempo (17). No entanto, nessa mesma coorte, ao considerar o comportamento desde a infância até a adolescência (2, 5, 8, 10 e 14 anos), avaliando os mesmos comportamentos, os autores identificaram que os amamentados por 6 meses ou mais apresentavam menor chance de apresentar comportamentos externalizantes do que os amamentados por menos de 6 meses (10).

Diversos estudos prospectivos investigaram a relação entre amamentação e hiperatividade. Uma coorte de nascimentos que acompanhou 9 525 crianças do Reino Unido até os 5 anos de idade evidenciou que aqueles amamentados por 2 a 4 meses apresentavam menor chance de desenvolver hiperatividade (OR = 0,65; IC95%: 0,43 a 0,99) quando comparados aos que nunca foram amamentados. Porém, tal associação não foi observada nas categorias de maior tempo de amamentação (12). Entretanto, Julvez et al. (28) observaram que as crianças de dois locais na Espanha que haviam sido amamentadas por 28 semanas tiveram redução no risco de hiperatividade (RR = 0,48; IC95%: 0,25 a 0,94) aos 4 anos em relação às amamentadas por menos de 2 semanas. Contrariamente, em outros estudos tal associação não foi observada na infância (13–15) e na adolescência (16).

**TABELA 1.** Características dos participantes da Coorte de Nascimentos de Pelotas de 1993, com informações completas sobre amamentação e comportamento nos acompanhamentos aos 4, 11 e 15 anos e comparação com a coorte original, Pelotas (RS), Brasil, 1993 a 2008^a^

Variável	Coorte original (n = 5 249) No. (%)	4 anos (n = 630)^b^ No. (%)	Valor *P*^c^	11 anos (n = 1 227) No. (%)	Valor *P*^c^	15 anos (n = 1 199) No. (%)	Valor *P*^c^
Renda familiar (quintis)			0,227		0,478		0,549
1 (menor)	1 030 (20,0)	113 (18,2)		238 (19,4)		237 (19,8)	
2	1 195 (23,3)	145 (23,4)		300 (24,4)		289 (24,0)	
3	889 (17,3)	129 (20,7)		232 (18,9)		227 (19,0)	
4	1 002 (19,5)	110 (17,7)		230 (18,8)		224 (18,7)	
5 (maior)	1 021 (19,9)	124 (20,0)		227 (18,5)		222 (18,5)	
Escolaridade da mãe (anos)			0,706		0,705		0,878
0 a 4	1 467 (28,0)	171 (27,2)		341 (27,4)		331 (27,3)	
5 a 8	2 425 (46,3)	284 (45,3)		596 (47,9)		577 (47,5)	
9 a 11	923 (17,6)	114 (18,1)		213 (17,1)		211 (17,4)	
≥ 12	427 (8,1)	59 (9,4)		93 (7,5)		95 (7,8)	
Idade da mãe (anos)			0,009		0,177		0,165
< 20	916 (17,4)	84 (13,3)		185 (14,9)		178 (14,6)	
20 a 24	1 447 (27,6)	158 (25,1)		354 (28,4)		340 (27,9)	
25 a 29	1 353 (25,8)	168 (26,7)		320 (25,7)		319 (26,3)	
30 a 34	956 (18,2)	140 (22,2)		251 (20,1)		243 (19,9)	
≥ 35	576 (11,0)	80 (12,7)		136 (10,9)		137 (11,3)	
Marido/parceiro vivendo com a família			0,171		0,177		0,088
Sim	4 600 (87,6)	564 (89,5)		1 109 (89,0)		1 088 (89,4)	
Não	649 (12,4)	66 (10,5)		137 (11,0)		129 (10,6)	
Tabagismo na gestação			0,193		0,792		0,693
Sim	1 752 (33,4)	194 (30,7)		411 (33,0)		399 (32,8)	
Não	3 497 (66,6)	436 (69,3)		835 (67,0)		818 (67,2)	
Consumo de álcool na gestação			0,858		0,787		0,897
Sim	267 (5,1)	31 (5,0)		61 (4,9)		63 (5,1)	
Não	4 983 (94,9)	599 (95,0)		1 184 (95,1)		1 154 (94,9)	
Idade gestacional (semanas)			0,083		0,290		0,242
< 37	611 (11,8)	59 (9,5)		133 (10,7)		128 (10,5)	
≥ 37	4 583 (88,2)	567 (90,5)		1 107 (89,3)		1 083 (89,5)	
Sexo			0,834		0,914		0,828
Masculino	2 606 (49,7)	310 (49,2)		616 (49,5)		600 (49,3)	
Feminino	2 643 (50,3)	320 (50,8)		629 (50,5)		617 (50,7)	
Peso de nascimento (g)			0,960		0,634		0,640
< 2 500	510 (9,7)	61 (9,6)		127 (10,2)		124 (10,2)	
≥ 2 500	4 723 (90,3)	569 (90,4)		1 119 (89,8)		1 093 (89,8)	
Duração da amamentação (meses)			0,852		0,919		0,962
< 1	241 (17,0)	101 (16,1)		202 (16,2)		199 (16,4)	
1 a 2,9	347 (24,5)	155 (24,6)		307 (24,7)		305 (25,0)	
3 a 5,9	330 (23,3)	141 (22,3)		299 (24,0)		287 (23,6)	
≥ 6	499 (35,2)	233 (37,0)		437 (35,1)		426 (35,0)	

^a^ O número considerado em cada acompanhamento pode diferir do número total do acompanhamento devido a perdas de acompanhamento ou de informação.

^b^ Todos os dados foram ponderados considerando o procedimento amostral utilizado aos 12 meses e 4 anos.

^c^ Teste do qui-quadrado de heterogeneidade.

Entre os mecanismos apontados na literatura para explicar a relação entre aleitamento materno e problemas de comportamento está a composição do leite materno, que, por ser rico em ácidos graxos poli-insaturados de cadeia longa, promove a formação das células cerebrais e favorece o bom desenvolvimento neurológico (neuroproteção e neurotransmissão) da criança (29, 30). Estudos mostraram que a maior concentração de ácidos graxos (ácidos graxos poli-insaturados de cadeia longa, ácido docosaexaenoico e ácido araquidônico) na mãe ou no cordão umbilical está associada com menor risco de ter hiperatividade, problemas de comportamento total e alguns problemas emocionais na infância (31, 32). Além disso, a interação entre a mãe e a criança, propiciada pelo contato constante e íntimo durante o período de amamentação, poderia trazer benefícios no comportamento futuro (33). Alguns estudos relataram que crianças amamentadas apresentavam, na infância, menos ansiedade, depressão, queixas somáticas, comportamentos internalizantes (34) e melhor desenvolvimento mental (30) e nas relações sociais (35). No entanto, deve-se considerar ainda que as características do aleitamento materno poderiam ser possivelmente um marcador de outros aspectos — individuais e familiares — não mensurados no presente estudo e que podem influenciar os problemas de comportamento, tais como história familiar de problemas de comportamento, relação conflituosa entre mãe e filho (33) e entre os pais (35, 36) e eventos negativos da vida (36), entre outros fatores.

**TABELA 2.** Duração da amamentação em relação a variáveis socioeconômicas, demográficas e comportamentais, Coorte de Nascimentos de Pelotas, 1993-1994, Pelotas (RS), Brasil

Variável	Duração da amamentação (meses)				Valor *P*^a^
	< 1 No. (%)	1 a 2,9 No. (%)	3 a 5,9 No. (%)	≥ 6 No. (%)	
Renda familiar (quintis)					0,923
1 (menor)	55 (19,4)	73 (25,7)	60 (21,1)	96 (33,8)	
2	61 (17,8)	93 (27,1)	82 (23,9)	107 (31,2)	
3	49 (19,3)	61 (24,0)	59 (23,2)	85 (33,5)	
4	56 (21,5)	73 (28,0)	57 (21,8)	75 (28,7)	
5 (maior)	49 (19,2)	57 (22,4)	61 (23,9)	88 (34,5)	
Escolaridade da mãe (anos)					0,107
0 a 4	86 (21,3)	105 (26,0)	88 (21,8)	125 (30,9)	
5 a 8	124 (18,7)	178 (26,9)	158 (23,8)	203 (30,6)	
9 a 11	57 (23,3)	54 (22,0)	51 (20,8)	83 (33,9)	
≥ 12	13 (12,4)	21 (20,0)	25 (23,8)	46 (43,8)	
Idade da mãe (anos)					< 0,001
< 20	58 (24,8)	72 (30,8)	53 (22,6)	51 (21,8)	
20 a 24	66 (17,3)	96 (25,2)	90 (23,6)	129 (33,9)	
25 a 29	92 (24,7)	86 (23,1)	87 (23,4)	107 (28,8)	
30 a 34	42 (15,3)	75 (27,4)	52 (19,0)	105 (38,3)	
≥ 35	22 (13,8)	29 (18,2)	41 (25,8)	67 (42,2)	
Marido/parceiro vivendo com a família					0,153
Sim	248 (20,0)	301 (24,2)	286 (23,0)	408 (32,8)	
Não	32 (18,1)	57 (32,2)	37 (20,9)	51 (28,8)	
Tabagismo na gestação		0,005			
Sim	103 (19,8)	157 (30,1)	115 (22,1)	146 (28,0)	
Não	177 (19,7)	201 (22,4)	208 (23,1)	313 (34,8)	
Consumo de álcool na gestação					0,381
Sim	13 (19,1)	21 (30,9)	18 (26,5)	16 (23,5)	
Não	267 (19,7)	337 (24,9)	305 (22,6)	443 (32,8)	
Idade gestacional	< 0,001				
< 37	87 (29,6)	81 (27,6)	60 (20,4)	66 (22,4)	
≥ 37	190 (17,0)	274 (24,5)	262 (23,4)	392 (35,1)	
Sexo					0,076
Masculino	146 (21,3)	184 (26,9)	153 (22,4)	201 (29,4)	
Feminino	134 (18,2)	174 (23,6)	170 (23,1)	258 (35,1)	
Peso de nascimento (g)					< 0,001
< 2 500	124 (29,5)	114 (27,1)	88 (21,0)	94 (22,4)	
≥ 2 500	156 (15,7)	241 (24,2)	235 (23,5)	365 (36,6)	

^a^ Teste do qui-quadrado de heterogeneidade.

É importante destacar algumas limitações deste estudo. Uma vez que dois diferentes instrumentos foram utilizados, não foi possível avaliar a trajetória dos problemas de comportamento da infância à adolescência. Porém, o fato de que o SDQ possui propriedades psicométricas adequadas e comparáveis à CBCL (32) e a alta sensibilidade de ambos para identificar problemas de comportamento em crianças e adolescentes (22, 24, 25) minimiza o viés de classificação. A utilização de subamostra nos acompanhamentos do 1o e 4o anos de vida é outra limitação deste estudo, uma vez que o pequeno tamanho amostral reduz o poder estatístico para detectar possíveis associações. No entanto, a subamostra apresentou características socioeconômicas e demográficas semelhantes à coorte original. Além disso, a fim de considerar a sobreamostragem de nascidos com baixo peso, foi utilizada a ponderação em todas as análises. Outras limitações foram a impossibilidade de estudar a associação entre os desfechos investigados no presente trabalho e o aleitamento materno exclusivo em razão de sua baixa prevalência na presente coorte, e a ausência de ajuste para alguns fatores de confusão considerados importantes, tais como a relação entre a mãe e o filho e a saúde mental materna, os quais poderiam reduzir as medidas de efeito se incluídos.

**TABELA 3.** Escore médio dos comportamentos externalizantes aos 4, 11 e 15 anos de acordo com variáveis socioeconômicas, demográficas e de saúde, Coorte de Nascimentos de Pelotas, 1993 a 2008, Pelotas (RS), Brasil

Escore de comportamentos externalizantes^a^		
4 anos Média (DP)	11 anos Média (DP)	15 anos Média (DP)^b^
Renda familiar (quintis)	*P* = 0,567^c^	*P* < 0,001^c^	*P* < 0,001^c^
1 (menor)	54,73 (10,02)	7,90 (4,76)	6,82 (4,70)
2	54,35 (10,82)	7,22 (4,84)	6,29 (4,75)
3	54,26 (11,26)	7,08 (4,95)	6,42 (4,80)
4	54,98 (9,61)	6,34 (4,64)	5,76 (4,53)
5 (maior)	54,66 (10,14)	5,39 (4,22)	4,98 (4,22)
Escolaridade da mãe (anos)	*P* = 0,349^c^	*P* < 0,001^c^	*P* < 0,001^c^
0 a 4	56,38 (11,25)	7,62 (4,80)	6,67 (4,70)
5 a 8	53,6 3(10,09)	7,15 (4,82)	6,31 (4,75)
9 a 11	54,06 (10,34)	5,64 (4,36)	5,26 (4,28)
≥ 12	55,23 (9,45)	4,55 (3,94)	4,34 (4,02)
Idade da mãe (anos)	*P* < 0,001^c^	*P* < 0,001^c^	*P* < 0,001^c^
< 20	57,95 (9,49)	8,13 (4,92)	7,24 (4,79)
20 a 24	56,27 (9,78)	6,97 (4,78)	6,17 (4,74)
25 a 29	54,28 (10,53)	6,61 (4,75)	5,87 (4,56)
30 a 34	52,15 (10,70)	6,10 (4,44)	5,44 (4,37)
≥ 35	52,61 (10,80)	6,15 (4,63)	5,58 (4,55)
Marido/parceiro vivendo com a família	*P* = 0,002	*P* < 0,001	*P* < 0,001
Sim	54,14 (10,40)	6,69 (4,73)	5,98 (4,62)
Não	58,41 (10,13)	7,85 (4,92)	6,85 (4,80)
Tabagismo na gestação	*P* < 0,001	*P* < 0,001	*P* < 0,001
Sim	57,64 (10,60)	7,76 (4,68)	7,09 (4,79)
Não	53,24 (10,10)	6,36 (4,80)	5,58 (4,49)
Consumo de álcool na gestação	*P* = 0,096	*P* = 0,002	*P* < 0,001
Sim	57,63 (9,99)	7,79 (4,93)	7,44 (5,06)
Não	54,43 (10,45)	6,78 (4,75)	6,00 (4,62)
Idade gestacional	*P* = 0,106	*P* = 0,170	*P* = 0,219
< 37	56,73 (9,90)	7,12 (4,89)	6,35 (4,59)
≥ 37	54,43 (10,47)	6,80 (4,75)	6,07 (4,66)
Sexo	*P* = 0,382	*P* < 0,001	*P* < 0,001
Masculino	54,22 (9,91)	7,49 (4,81)	6,38 (4,68)
Feminino	54,95 (10,96)	6,19 (4,63)	5,79 (4,61)
Peso de nascimento (g)	*P* = 0,281	*P* = 0,051	*P* = 0,265
< 2 500	55,97 (9,61)	7,27 (4,87)	6,33 (4,72)
≥ 2 500	54,44 (10,53)	6,78 (4,75)	6,05 (4,64)
Duração da amamentação (meses)	*P* = 0,070^c^	*P* = 0,009^c^	*P* = 0,137^c^
< 1	56,17 (9,35)	7,07 (4,92)	6,08 (4,66)
1 a 2,9	54,89 (10,57)	7,08 (4,75)	6,08 (4,85)
3 a 5,9	55,31 (10,20)	6,78 (4,74)	6,08 (4,59)
≥ 6	53,23 (10,85)	6,28 (4,86)	5,57 (4,41)

^a^ Maior escore representa pior resultado no teste (escore ≥ 69 aos 4 anos e escore ≥ 14 aos 11 e 15 anos representam comportamento anormal).

^b^ Valor *P* obtido pelo teste do qui-quadrado de heterogeneidade.

^c^ Teste do qui-quadrado de tendência linear.

Entre os pontos fortes do presente estudo, vale mencionar que os resultados foram pouco suscetíveis a viés de memória, uma vez que a informação sobre aleitamento materno foi coletada aos 12 meses, sendo curto o período recordatório entre a exposição e a obtenção do dado. Ademais, a utilização do aleitamento materno em quatro categorias permitiu explorar a possibilidade de efeito dose-resposta, pouco avaliado pelos estudos encontrados sobre a temática. Outro ponto positivo foi a aplicação do instrumento de avaliação do comportamento à mãe/responsável, pois sua aplicação ao adolescente poderia subestimar a prevalência dos problemas de comportamento, especialmente externalizantes (36).

Os resultados sugerem que a amamentação por pelo menos 6 meses reduz o risco de hiperatividade aos 11 anos, mas não aos 15 anos. Muito embora os resultados tenham sido divergentes nas idades investigadas, a recomendação do aleitamento materno deve ser mantida por suas diversas vantagens para o desenvolvimento infantil. Novos estudos longitudinais poderão considerar em suas análises os múltiplos fatores que influenciam os comportamentos externalizantes (presença do pai no ambiente familiar, violência doméstica e maus-tratos) e a qualidade da relação mãe-filho (tipo de vínculo e suporte emocional), para explorar de modo mais específico essa relação e suas consequências no comportamento durante a infância e adolescência.

## Agradecimentos.

Este artigo foi realizado com dados do estudo “Coorte de Nascimentos de Pelotas, em 1993“, conduzido por professores pesquisadores do Programa de Pós-Graduação em Epidemiologia da Universidade Federal de Pelotas (UFPel), com apoio da Associação Brasileira de Saúde Coletiva (ABRASCO). De 2004 a 2013, a Coorte de Nascimentos de 1993 foi financiada pelo Wellcome Trust, Conselho Nacional de Desenvolvimento Científico e Tecnológico (CNPq) e Fundação de Amparo à Pesquisa do estado de Rio Grande do Sul (FAPERGS). Fases anteriores do estudo foram financiadas pela União Europeia, Programa de Apoio a Núcleos de Excelência (PRONEX), Pastoral da Criança, CNPq e Ministério da Saúde.

## Declaração.

As opiniões expressas no manuscrito são de responsabilidade exclusiva dos autores e não refletem necessariamente a opinião ou política da *RPSP/PAJPH* ou da Organização Pan-Americana de Saúde (OPAS).

**TABELA 4.** Análise bruta e ajustada da associação entre amamentação e comportamentos externalizantes na infância e adolescência, Coorte de Nascimentos de Pelotas, 1993 a 2008

Duração da amamentação (meses)	Bruta		Ajustada^a^	
	RR^b^	IC95%	RR^b^	IC95%
4 anos (*Child Behavior Checklist*)					
Comportamentos externalizantes	< 1	Referência		Referência	
	1 a 2,9	0,83	0,38 a 1,83	0,90	0,40 a 2,04
	3 a 5,9	0,94	0,43 a 2,07	1,03	0,45 a 2,32
	≥ 6	0,70	0,33 a 1,49	0,78	0,36; 1,70
Comportamento delinquente	< 1	Referência		Referência	
	1 a 2,9	1,06	0,51 a 2,21	1,15	0,54 a 2,46
	3 a 5,9	0,76	0,33 a 1,71	0,80	0,34 a 1,87
	≥ 6	0,81	0,39 a 1,67	0,90	0,42 a 1,90
Comportamento agressivo	< 1	Referência		Referência	
	1 a 2,9	1,00	0,44 a 2,23	1,10	0,48 a 2,55
	3 a 5,9	0,99	0,43 a 2,26	1,07	0,45 a 2,55
	≥ 6	0,80	0,37 a 1,75	0,90	0,40 a 2,04
11 anos (*Strengths and Difficulties Questionnaire*)					
Comportamentos externalizantes	< 1	Referência		Referência	
	1 a 2,9	0,85	0,51 a 1,43	0,91	0,53 a 1,54
	3 a 5,9	0,86	0,50 a 1,45	0,92	0,54 a 1,59
	≥ 6	0,73	0,44 a 1,21	0,78	0,46 a 1,32
Hiperatividade	< 1	Referência		Referência	
	1 a 2,9	0,70	0,42 a 1,16	0,71	0,43 a 1,19
	3 a 5,9	0,80	0,49 a 1,32	0,76	0,45 a 1,28
	≥ 6	0,55	0,34 a 0,91	0,54	0,32 a 0,91
Problemas de conduta	< 1	Referência		Referência	
	1 a 2,9	1,19	0,72 a 1,98	1,37	0,80 a 2,35
	3 a 5,9	1,06	0,62 a 1,80	1,19	0,68 a 2,09
	≥ 6	1,22	0,75 a 1,97	1,41	0,84 a 2,36
15 anos (*Strengths and Difficulties Questionnaire*)					
Comportamentos externalizantes	< 1	Referência		Referência	
	1 a 2,9	0,85	0,51 a 1,41	0,98	0,57 a 1,67
	3 a 5,9	0,79	0,46 a 1,34	0,90	0,52 a 1,58
	≥ 6	0,68	0,41 a 1,13	0,79	0,47 a 1,35
Hiperatividade	< 1	Referência	Referência		
	1 a 2,9	0,86	0,48 a 1,54	0,90	0,50 a 1,64
	3 a 5,9	0,85	0,46 a 1,55	0,88	0,48 a 1,64
	≥ 6	0,64	0,36 a 1,17	0,66	0,36 a 1,21
Problemas de conduta	< 1	Referência		Referência	
	1 a 2,9	1,10	0,66 a 1,82	1,20	0,71 a 2,03
	3 a 5,9	0,98	0,58 a 1,67	1,07	0,62 a 1,87
	≥ 6	0,72	0,43 a 1,22	0,81	0,47 a 1,39

^a^ Ajuste para renda familiar, escolaridade da mãe, idade da mãe, mãe com marido ou parceiro vivendo com a família, tabagismo na gestação, consumo de álcool na gestação, idade gestacional, sexo e peso de nascimento.

^b^ RR: Razão de risco proveniente da regressão de Poisson.
